# Influence of Proximal-Cervical Undermined Enamel Areas on Marginal Quality and Enamel Integrity of Laboratory and CAD/CAM Ceramic Inlays and Partial Crowns

**DOI:** 10.3390/jfb16030082

**Published:** 2025-03-01

**Authors:** Roland Frankenberger, Katharina Friedrich, Marie-Christine Dudek, Julia Winter, Norbert Krämer, Matthias J. Roggendorf

**Affiliations:** 1 Department of Operative Dentistry, Endodontics, and Pediatric Dentistry, Medical Center for Dentistry, University Medical Center Giessen and Marburg, 35039 Marburg, Germanymatthias.roggendorf@staff.uni-marburg.de (M.J.R.); 2 Department of Paediatric Dentistry, Medical Center for Dentistry, University Medical Center Giessen and Marburg, Campus Giessen, Schlangenzahl 14, 35392 Giessen, Germany; julia.winter@staff.uni-marburg.de (J.W.); norbert.kraemer@dentist.med.uni-giessen.de (N.K.)

**Keywords:** inlay, partial crown, ceramic, adhesive, self-etch, etch-and-rinse, marginal quality, cracking

## Abstract

(1) The aim of this in vitro study was to investigate the handling of proximal-cervical undermined enamel margins on the adhesive performance of differently fabricated and differently cemented ceramic inlays and partial crowns (2) Methods: 192 extracted third molars received MOD (*n* = 96) and partial crown (*n* = 96) preparations. A mesial 2 × 2 × 4 mm cervical groove was created in dentin to simulate a deeper (dentin) caries excavation. This dentin groove was either left (G/groove), filled with composite (F/filling), or completely removed (D/dentin). Distal proximal boxes did not receive a groove and served as controls within the same tooth. Labside (e.max Press) restorations additionally went through a temporary phase. Labside and chairside (e.max CAD) inlays and partial crowns were then adhesively luted with Syntac/Variolink Esthetic (SV) or Adhese Universal/Variolink Esthetic (AV). Initially, and again after thermomechanical loading (TML: 1 million cycles at 50 N, 25,000 thermocycles at 5 °C/55 °C), specimens were molded and the resulting 24 groups of epoxy replicas (*n* = 8) were gold-sputtered and examined for marginal gaps using scanning electron microscopy (200× magnification). Light microscopy (10× magnification) was used to measure proximal cervical crack propagation in adjacent enamel. (3) Results: Regardless of the adhesive system, D groups generally showed significantly lower marginal quality (79–88%; *p* < 0.05), with the universal adhesive performing better than the multi-step adhesive system (*p* < 0.05). Subgroups G and F were similar in marginal quality (94–98%; *p* > 0.05) and not worse than the controls (*p* > 0.05) regardless of the adhesive system, but showed less cracking in F than in G (*p* < 0.05). In general, fewer cracks were observed in chairside CAD/CAM restorations than in laboratory-fabricated restorations (*p* < 0.05). Partial crowns showed better marginal quality (96–98%) and less cracking than inlays (*p* < 0.05). (4) Conclusions: If the dentin level is lower than the enamel level in ceramic preparations after caries excavation in the proximal box, the resulting undermined enamel should not be removed. In terms of enamel integrity, partial crowns outperformed inlays.

## 1. Introduction

**Figure 1:**
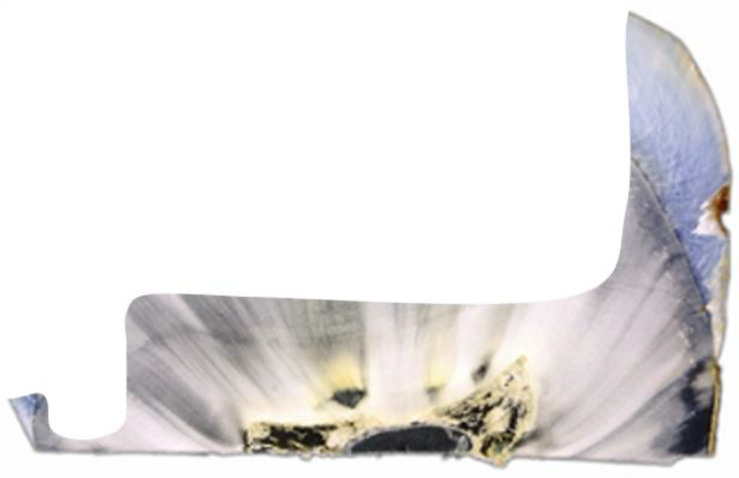
Undermined proximal cervical area (groove) in the histologic preparation.

Ceramic inlays and partial crowns are a globally recognized prime type of restoration with clinically excellent survival rates [1,2,3,4,5,6]. This applies equally to chairside work and CAD/CAM restorations [1,2,3,4,5,6]. However, clinical problems still lie in the technique sensitivity of the adhesive systems [7,8,9]; the selection of the polymerization mode of the adhesive and luting composite [10,11,12]; and an accurate, ceramic-compatible preparation with smooth cavity geometries avoiding tensile forces in ceramics [12]. The latter depends not only on the tooth structure that can be adhesively stabilized but also on the extent of dentin caries to be excavated. The proximal cavity geometry after caries excavation is usually clinically irregular and, in many cases, vertically deeper in dentin than in enamel, resulting in characteristic undermined enamel lamellae (Figure 1).

Also, when selective dentin caries excavation is practiced, which is generally scientifically recommended today [13], the majority of the carious biomass is still removed, resulting in the same phenomenon. When looking at the literature in the field of adhesive ceramic restorations, it is surprising that the problem presented in this study has never been investigated, as it is a phenomenon that certainly occurs millions of times in daily practice. It is obviously a rather trivial problem, but of considerable clinical relevance for daily routine.

**Figure 2:**
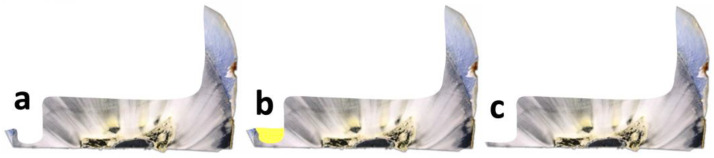
Undermined proximal-cervical area (groove) in the histological preparation and different ways of dealing with it: (**a**) leave in place (G groups/groove); (**b**) fill with composite (F groups/filling); (**c**) remove the lamella (D groups/dentin).

For the involved dentist, three completely different clinical scenarios are possible for the frequently occurring case of the described pronounced undermined enamel clasp: the cavity is left as is and the enamel remains undermined there until adhesive cementation (Figure 2a), the dentin groove is filled adhesively with directly applied resin composite (Figure 2b), or the enamel lamella is completely removed in this area (Figure 2c), which would hypothetically prevent the risk of fracture or cracking not only later but also potentially under load when the temporary restoration is in place for several days; however, this almost always leads to complete proximal-cervical enamel loss and the restoration ending in dentin/cement.

The aim of the present in vitro study was to investigate these described pre-preparative restorative scenarios with regard to their influence on adhesive performance of ceramic inlays and partial crowns, whereby the type of fabrication as well as marginal quality on the one hand and crack formation in enamel on the other hand were to be examined. In addition to the cavity geometry, different luting systems were also considered, i.e., a multi-bottle system (Syntac, Ivoclar, Schaan, Principality of Liechtensein) and a universal adhesive (Adhese Universal, Ivoclar) were combined with a dual-curing luting composite (Variolink Esthetic DC, Ivoclar).

The four null hypotheses of the study were that (1) the handling of the enamel lamella would have no effect on the adhesive performance (margin and enamel cracks), (2) that the luting system would have no effect on the different groups, (3) that there would be no difference between inlays and partial crowns, and (4) that there would be no difference between chairside and labside ceramics.

## 2. Materials and Methods

In oder to address the proposed research question, thermomechanical loading (i.e., chewing simulation) was used in vitro to simulate the clinical situation of ceramic restorations under load. The primary outcome variable was marginal quality in enamel and dentin; the secondary outcome variable was crack formation in adjacent enamel. Therefore, 192 caries-free human wisdom teeth of similar size (max. 3 mm difference in height and 2 mm for both mesio-distal and bucco-oral) with >80% developed and undamaged roots, freshly extracted for therapeutic reasons, were included in the study and randomly assigned to 24 groups (Research randomizer, Table 1). All patients had given written consent for their teeth to be used for scientific purposes, the study was conducted in accordance with the Helsinki Declaration as well as following the CONSORT rules for in vitro research, and the study protocol was reviewed and approved by a local ethics committee (University of Giessen, project code 143/09). Teeth specimens were stored in 0.5% chloramine-T for a maximum of 30 days, cleaned, and examined for cracks by light microscopy (10× magnification). The minimum sample size was calculated to be *n* = 8 in each group (PASS 15 according to previous studies [14,15,16], with a 95% confidence interval, an 80% study power, a standard deviation of 3, and a 2-unit difference between control and test groups) in terms of mean percentage of gap-free margins and mean total crack length in enamel.

**Figure 3:**
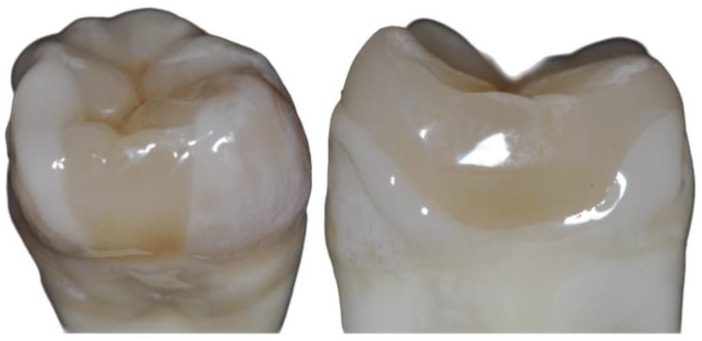
Inlay vs. partial crown in vitro (here: labside procedure).

The teeth specimens received standardized MOD cavities with a proximal box level 2 mm above the amelo-cemental junction, 50% of which were further prepared into partial crowns with a vertical cusp thickness of 1.5 mm (Figure 3; Table 1). Cavities were prepared using a special ceramic set (expert set, Komet, Lemgo, Germany) in a handpiece with 3 cooling nozzles and a flow rate of 30 mL/min. Cavity sizes were standardized using a ruler under 10× magnification. Inner angles were rounded and the edges were not beveled. A 2 × 2 × 4 mm groove was prepared in the mesial box to simulate the often deeper caries excavation there (see Figure 1), resulting in a 2 mm mesio-distal lamella of undermined enamel. This groove was either left in place (G) or filled directly with composite (F; Adhese Universal self-etch, Ivoclar Vivadent, Schaan, Liechtenstein and SDR, Dentsply Sirona, Konstanz, Germany, one layer). All light-curing processes were carried out with a Bluephase polymerization light (Ivoclar Vivadent), during which the light intensity was continuously checked with a radiometer (Demetron Research Corp., Danbury, CT, USA) to ensure that 1000 mW/cm^2^ was always reliably exceeded.

In group F, after the adhesive build-up was completed, finishing was carried out with the above-mentioned diamond bur to expose the enamel margins again; the composite areas were sandblasted for 5 s (Rondoflex 360 plus, KaVo, Biberach, Germany).

Conventional (Flexitime, Kulzer-Dental, Hanau, Germany) or optical (Omnicam, Cerec, Dentsply Sirona, Bensheim, Germany) impressions were taken of the cavities and chairside and labside ceramic inlays and partial crowns (e.max CAD/e.max Press, Ivoclar Vivadent) were fabricated, the latter having been manually produced on stone dies (Fuji Rock, GC, Tokio, Japan). The ceramic intaglio surface was pretreated with 5% hydrofluoric acid (Vita Ceramic Etch, Vita Zahnfabrik, Bad Säckingen, Germany) for 20 s. After spraying and drying, silane (Monobond Plus, Ivoclar) was applied for 60 s and no additional adhesive was applied. The labside restorations received temporary inlays and partial crowns (Luxatemp, DMG, Hamburg, Germany), which were cemented with TempGrip (Dentsply Sirona) and subjected to a shortened TML (1000 × 50 N and 25 thermocycles). The final restorations were adhesively cemented with Syntac or Adhese Universal (Ivoclar, both under etch-and-rinse conditions with 15 s dentin and 30 s enamel etching) and Variolink Esthetic DC according to the manufacturer’s instructions, during which the adhesive was polymerized separately in the cavity with Adhese Universal (20 s) and not with Syntac. Variolink Esthetic DC was polymerized for 20 s per side, a total of 120 s per restoration for Adhese Universal and 240 s for Syntac. The restoration margins were finished with Soflex discs (Solventum, Seefeld, Germany) in three decreasing grit sizes.

**Figure 4:**
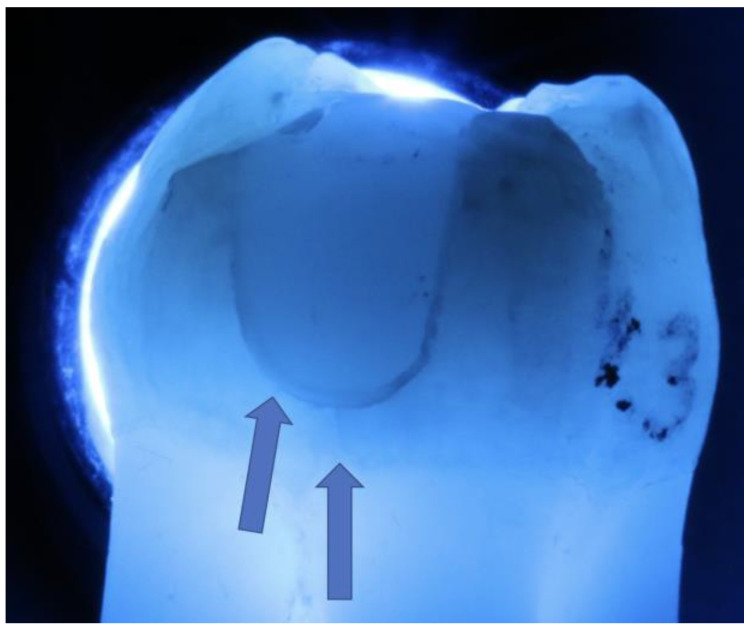
Proximal-cervical cracks (arrows) after TML under light transmission.

Directly after adhesive cementation (chairside restorations) or directly after provisional restoration (labside restorations) and after TML, the total length of enamel cracks below the distal (control) and mesial proximal boxes on the original samples was measured by light microscopy (test groups G/F only; image: Z6 APO, Leica, Wetzlar, Germany/10× under transmission; measurement: Photoshop CS6 2024, Adobe Systems Software Ireland Limited, Dublin, Ireland; Figure 4).

**Figure 5:**
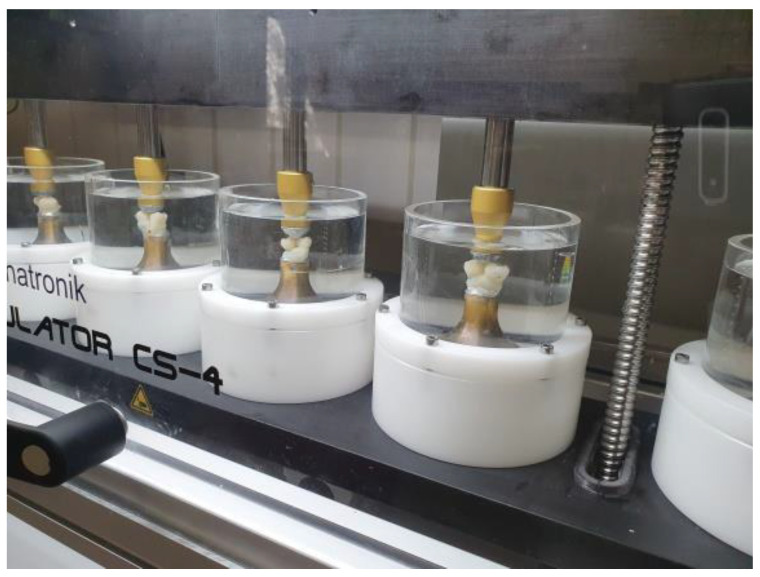
Chewing simulation: 1 million cycles of 50 N each, arranged in pairs.

After 21 days of storage in distilled water at 37 °C, the first impression was taken of the specimens (Flexitime) and a first set of epoxy resin replicas (Alpha-Die, Schütz Dental, Rosbach, Germany) was fabricated. Thermomechanical loading (TML) of the teeth specimens was performed in a chewing simulator (CS4 professional line, SD Mechatronik, Feldkirchen, Germany) under water. No artificial saliva or similar was used. Two teeth specimens were installed in a chewing simulator chamber in such a way that its 6 mm steatite antagonist “chewed” two distal marginal ridges (secured with occlusion foil). The total number of mechanical cycles was 1 million at 50 N at a frequency of 0.5 Hz. Prior to this, the specimens underwent 25,000 thermal cycles between 5 °C and 55 °C (30 s immersion time, THE 1100, SD Mechatronics; Figure 5).

**Figure 6:**
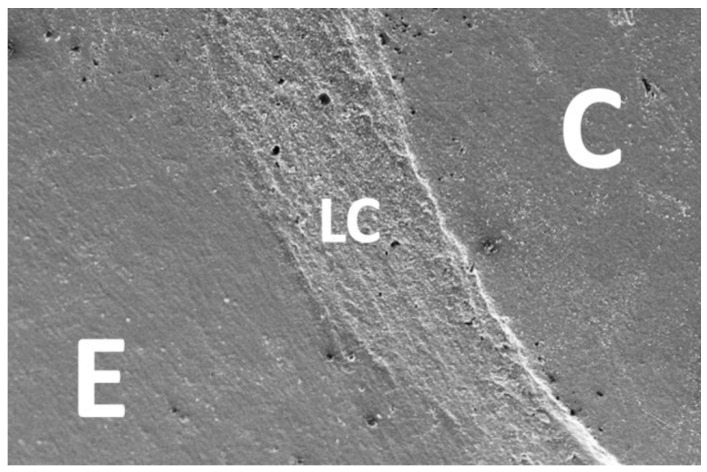
Representative SEM image: C: ceramic, LC: luting composite, E: enamel.

**Figure 7:**
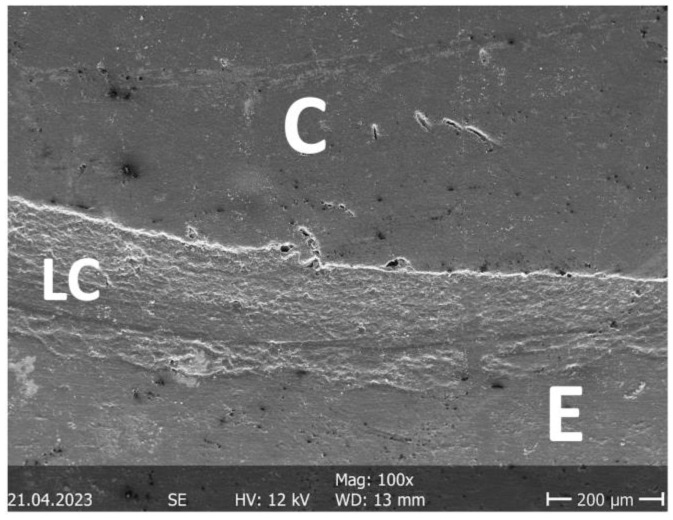
SEM image of the marginal quality measurement (100×). C: ceramic, LC: luting composite, E: enamel. Although the image shows slight irregularities, it is completely free of gaps. The majority of the specimens examined exhibited very good marginal quality throughout. Only in dentin was this not the case in some cases (Figure 10).

**Figure 8:**
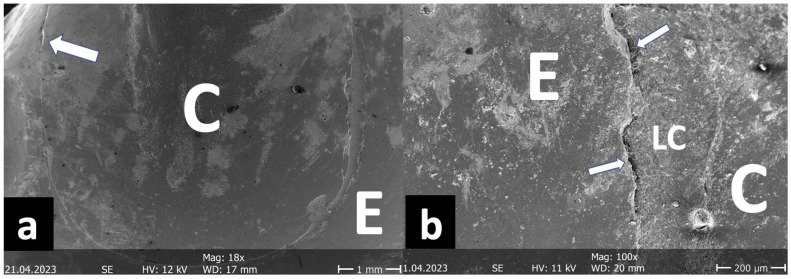
SEM image of the marginal quality measurement ((**a**) 18×/(**b**) 100×) from the group Inlay/Chairside/G/Syntac. C: ceramic, LC: luting composite, E: enamel. The image shows that, after TML, the enamel margins were almost exclusively defective in the upper area of the proximal box (arrows).

**Figure 9:**
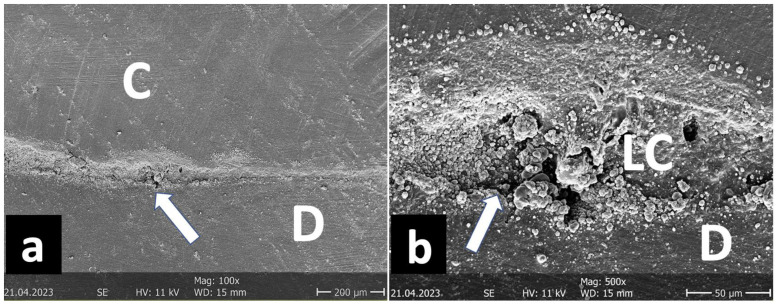
SEM image of the marginal quality measurement ((**a**) 100×/(**b**) 500×). C: ceramic, LC: luting composite, D: dentin. Group Inlay/Labside/D/Syntac. The image shows mechanical disintegration between dentin and luting composite with exposure of individual fillers (arrows).

After TML, the next crack measurement was carried out and the second set of replicas was prepared. Both sets of expoxy replicas were sputter coated with gold/palladium (Polaron SC502, Fisons Instruments, Ipswich, UK) and analyzed under a scanning electron microscope (Phenom, FEI, Amsterdam, The Netherlands) at 200× magnification. The marginal quality of the different interfaces (enamel luting composite, dentin luting composite, ceramic luting composite) was divided into “gap-free”, “gap/irregularity”, and “not assessable” and classified as a percentage of the gap-free margin in relation to the total margin. For better illustration, further SEM images were taken at different magnifications (Figure 6, Figure 7, Figure 8 and Figure 9).

Statistical analysis was performed using SPSS 17 (SPSS Inc., Chicago, IL, USA). After testing the non-normal distribution (Kolmogorov–Smirnov test), significant changes over time were calculated using the Wilcoxon test and differences between the groups were calculated using the Mann–Whitney U test at a significance level of 5%.

## 3. Results

### 3.1. Marginal Quality

**Figure 10:**
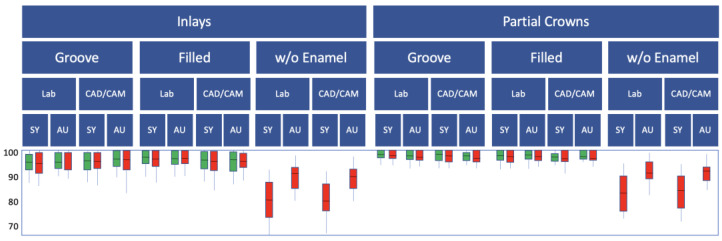
Results of the marginal quality (% gap-free enamel margin in the boxplot diagram with control/green and test groups/red, for the dentin groups only dentin margins; SY: Syntac; AU: Adhese Universal).

The results for SEM marginal quality evaluation are shown in Figure 10.

As the proportion of gap-free margins was initially 99–100% in all groups, the data are not shown for reasons of clarity and to avoid confusion for readers. Regardless of the adhesive system, marginal quality significantly dropped in all groups after TML (*p* < 0.05; Wilcoxon test; Figure 10). Also independent of the adhesive system used, D groups generally showed significantly lower marginal quality (*p* < 0.05; Mann–Whitney U-test), with the universal adhesive performing better than the multi-step adhesive system (*p* < 0.05; Mann–Whitney U-test; Figure 10). Subgroups G and F were similar in marginal quality regardless of the adhesive system (*p* > 0.05; Mann–Whitney U-test; Figure 10) and not significantly worse than the controls (*p* > 0.05; Mann–Whitney U-test; Figure 10). Partial crowns showed better marginal qualities in the enamel areas than MOD inlays (*p* < 0.05; Mann–Whitney U-test; Figure 10); in the dentin margins, the difference between inlays and partial crowns was not significant (*p* > 0.05; Mann–Whitney U-test; Figure 10).

### 3.2. Proximal Enamel Crack Propagation

**Figure 11:**
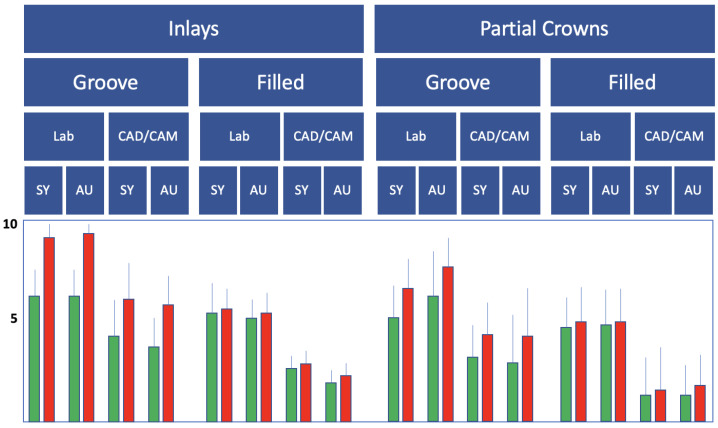
Results of the proximal-cervical crack increase (mm) in the bar chart with control/green and test groups/red; SY: Syntac; AU: Adhese Universal. The D groups are not listed, as no enamel was present in the proximal-cervical area in these groups. Error bars are only shown in the upper half for better visibility.

The evaluation of the proximal-cervical crack increase showed significantly less cracking at F than at G for both the inlays and the partial crowns (*p* < 0.05; Mann–Whitney U-test; Figure 11). In general, significantly fewer cracks were observed in CAD/CAM restorations than in laboratory-fabricated restorations (*p* < 0.05; Mann–Whitney U-test; Figure 11), with the greatest increase in cracking measured during the simulated provisional wearing time. Partial crowns showed fewer proximal enamel cracks than inlays (*p* < 0.05; Mann–Whitney U-test; Figure 11).

## 4. Discussion

Ceramic inlays and partial crowns represent one of the most successful clinical options for carious lesions in posterior teeth [1,2,3,4,5]. Compared to the early days, when ceramics suffered significant brittleness and were consequently quite prone to fractures [3,4,5], this is not the primary concern today. However, a relatively large window of contamination [6] as well as substantial technique sensitivity of adhesive luting procedures still exist [2].

On first sight, one is surprised that the investigated phenomenon has never been investigated because it is common in almost every proximal caries therapy. It is obviously a rather trivial problem, but of considerable clinical relevance for daily routine. Therefore, the aim of the present in vitro study was to investigate the consequences of different ways of managing proximal enamel lamellae; the null hypotheses were formulated in the introduction and all of them had to be rejected. Whether disregarding the procedure suggested here automatically leads to clinical failure cannot, in all honesty, be completely proven. For the ultimate proof, the randomized, clinical-prospective study still remains the most suitable instrument [2,4,7]. However, the latter is not ethically indicated in view of the experimental question at hand. Thermomechanical loading (TML/chewing simulation) is an established in vitro method for the preclinical evaluation of different restorative materials, especially when it is combined with replicas being investigated under an SEM [14,15]. TML represents a cyclic fatigue test that is very close to the clinical situation, where permanent, subcritical loads are generally far more common than events leading directly to failure. A special characteristic of our setup is that the antagonist ball simultaneously hits two lateral ridges—this is on the one hand very close to the clinical situation; on the other hand, it is not completely impossible that the load distribution is not 50:50 in any case. Compared to other studies, the total number of cycles in the present study may be very high with 1 million, but here too we tended to follow the majority of laboratory studies on causal simulation and therefore chose the worst-case scenario as well as facing the fact that the chosen 50 N may be on the lower end, especially in bruxism patients. In our opinion, a direct transferability of X cycles of TML to Y years of clinical exposure is not factual, but based on relevant in vitro–in vivo comparisons in the present study, one can assume a clinical equivalence period of approximately 10 years when facing the present TML scenario because, e.g., two pairs of in vitro vs in vivo studies have revealed a certain correlation in findings between 100,000 cycles in vitro and two years of clinical service in vivo [16].

In view of the decision to define the distal, not additionally prepared proximal boxes as “control groups”, at first glance this is a critical aspect of the present methodology, at least statistically. This definition could be viewed even more critically, since a one-tooth-to-two-tooth intercuspidation was simulated, as in the mouth, but the boxes under consideration were subjected to particular stress. However, a look at the results clearly shows that the mesial (test) and distal boxes (control) in the “good” groups did not differ significantly and therefore the chosen method of grouping appears acceptable. Furthermore, even with high standardization procedures in vitro, there may still be quite a variation in natural substrates—and this aspect was completely excluded using the present setup.

All three clinical scenarios investigated in the present in vitro study are plausible in the presence of proximally deeper dentin levels compared to the enamel, i.e., leaving the groove (G) and simply filling it with the luting composite later appears to be pragmatic, simple, and, at first glance, easy to solve with the adhesive technique without major effort. Nevertheless, this approach raises the question of whether these areas are at risk of fracture due to their lack of dentin support, e.g., during try-in or in the course of adhesive cementation, which certainly correlates with the extent of undermining, but would not be clinically acceptable in any case.

Another important factor in the course of crack evaluation is non-adhesive provisional restoration in laboratory-fabricated restorations. Therefore, both CAD/CAM restorations (without temporaries) and laboratory-fabricated restorations (with temporaries and a corresponding shortened chewing simulation) were included in the present study [15]. In the course of the present in vitro study, it was indeed shown that adhesive re-stabilization with the luting composite was not problematic in terms of marginal quality, but very problematic in terms of crack propagation, and this was much more pronounced in the laboratory-fabricated restorations. The time-limited chewing simulation of the provisionally restored teeth compared to the overall observation period was apparently sufficient to significantly influence the final result of crack propagation, which once again confirms the good clinical potential of chairside CAD/CAM restorations.

Even without the described findings from the G groups, we considered filling the described dentin groove with composite (F) to be an effective approach. In this case, a universal adhesive was bonded in self-etch mode and restored with an established bulk-fill composite. The disadvantage of this approach is traditionally that the enamel margins have to be refinished again for re-exposure, which means that the cervical extension of the preparation is always deeper than in the R groups. It was shown in all groups that this procedure was nevertheless significantly better than the other groups and can therefore also be recommended clinically.

It was generally interesting that marginal gaps in the enamel after TML were mainly found in the upper third of the proximal box (Figure 8) and not, as often postulated, in the cervical part. This phenomenon has already been described, in the 1990s by Mehl et al. [14]. The increase in cracks over time (before/after TML) was different; here, it was clear that mainly the proximal-cervical portion was affected by significant increases in cracks in the G groups. Finally, it was also interesting to note that both evaluated parameters (gap-free margins and crack increase) were better for the partial crowns than for the inlays, regardless of the cementation mode.

Finally, in the D groups, the undermined enamel clasp was completely removed, which meant that these restorations always ended up in the dentin/root cement. Although this technique would eliminate the cervical enamel cracks and the risk of fracture, the greater susceptibility to hydrolysis and enzymatic degradation in the dentin would be expected to result in more adhesive fatigue per se [17,18,19]. This suspicion was clearly confirmed by the results of the marginal quality examination: dentin margins showed significantly lower marginal quality than enamel margins in all groups, even if the measured proportions of gap-free margins in dentin were not catastrophic (Figure 9). The D group would only be a recommendable alternative if, for example, parts of the enamel had broken off during fitting or try-in or if the crack increase in the adjacent, non-dentin-supported enamel was very pronounced. In general, it can be said that fatigue of the composite–dentin bond is not an unsolvable problem today, but that restoration margins should not be placed in the root dentin without necessity if viable alternatives (groups G and F) are available [18].

A secondary result of the present study is the standardized evaluation of the effectiveness of different adhesive strategies in adhesive luting, which emerge from the test groups and provide additional, clinically relevant information for daily work. For example, the use of multi-bottle adhesives was a recognized gold standard, not least in the adhesive cementation of ceramics in tooth preservation. This term is derived from thousands of successful in vitro and in vivo studies, clearly documented low technique sensitivity and, last but not least, the presence of a hydrophobic bonding agent, which has been and is attested to be less susceptible to hydrolysis [20,21]. However, studies from the last decade in particular also show that the class of universal adhesives has caught up considerably in terms of overall adhesive performance and is no longer second-rate in its capacity as a single-bottle bonding agent [20,21]. In addition, they solve a major problem in the adhesive cementation of indirect restorations, as in the present study: compared to the rather highly viscous bonding agents of multi-bottle adhesives, where separate polymerization always entails the risk of not bringing the restoration into the final position during cementation, universal adhesives can easily be blown so thinly that they can be polymerized separately at any time [22,23]. This was also shown in the present study, as Heliobond was not polymerized separately with Syntac; the deep margins were also significantly worse compared to the universal adhesive groups despite 240 s light-curing. If the adhesive was not polymerized separately, it is possible that the evaluation of the margin still gives a positive picture, as the light source ensures sufficient polymerization at least at the margin. Even if the direct comparison with cervical fillings is somewhat limited, our results confirm the observations of other working groups with advantages for the universal adhesives compared to older multi-bottle adhesives, which have already been confirmed in vivo [24,25].

Last but not least, it should be stated that bonding to dentin, especially using indirect restorations with fewer polymerization shrinkage issues compared to resin composites, of course has good potential, and clinical observations e.g., with deep margin elevation are also promising [26,27,28,29]; however, based on our findings, removing enamel when it is not necessary should be avoided, because older clinical results have already shown good performance even when proximal enamel is very thin [30,31].

## 5. Conclusions

Within the limits of the present in vitro study, the following can be concluded:

- When the dentin level lays below the enamel level after proximal excavation, the resulting undermined enamel should not be removed;
- Instead, undermined enamel should be adhesively built up;
- The universal adhesive Adhese Universal outperformed the former gold standard, the multi-step adhesive Syntac;
- Regarding enamel integrity, ceramic partial crowns performed better than inlays.

## Figures and Tables

**Table 1:** Flowchart of the group classification (S: Syntac; A: Adhese Universal).

192 extracted third molars (*n* = 8)																							
96 MOD inlays												96 partial crowns											
Groove				Filled				w/o enamel				Groove				Filled				w/o enamel			
Lab		CAD/CAM		Lab		CAD/CAM		Lab		CAD/CAM		Lab		CAD/CAM		Lab		CAD/CAM		Lab		CAD/CAM	
S	A	S	A	S	A	S	A	S	A	S	A	S	A	S	A	S	A	S	A	S	A	S	A

## Data Availability

The original contributions presented in the study are included in the article, further inquiries can be directed to the corresponding author.
